# Effects of dietary supplementation of formaldehyde and crystalline amino acids on gut microbial composition of nursery pigs

**DOI:** 10.1038/s41598-018-26540-z

**Published:** 2018-05-25

**Authors:** H. E. Williams, R. A. Cochrane, J. C. Woodworth, J. M. DeRouchey, S. S. Dritz, M. D. Tokach, C. K. Jones, S. C. Fernando, T. E. Burkey, Y. S. Li, R. D. Goodband, R. G. Amachawadi

**Affiliations:** 1 Department of Animal Sciences and Industry, College of Agriculture, Kansas State University, Manhattan, 66506 USA; 2 Department of Diagnostic Medicine/Pathobiology, College of Veterinary Medicine, Kansas State University, Manhattan, 66506 USA; 3 Department of Animal Sciences, College of Agricultural Sciences and Natural Resources, University of Nebraska-Lincoln, Lincoln, 68527 USA; 4 Department of Clinical Sciences, College of Veterinary Medicine, Kansas State University, Manhattan, 66506 USA

## Abstract

Formaldehyde-based feed additives are approved in the US for *Salmonella* control and reducing bacterial contamination in animal feed. However, we hypothesize formaldehyde inclusion in swine diets may influence gut microbial composition due to its antimicrobial properties which might negatively influence microbial populations and pig growth performance. Also, formaldehyde inclusion in diets is known to reduce the dietary availability of amino acids. Therefore, our study was conducted to characterize if the effects of feed formaldehyde-treatment are due to influences on microbial population or diet amino acid (AA) sources. Dietary treatments were arranged in a (2 × 2) + 1 factorial with formaldehyde treatment (none vs. 1000 ppm formaldehyde) and crystalline AA inclusion (low vs. high) with deficient AA content plus a positive control diet to contain adequate AA content without dietary formaldehyde. Treating diets with formaldehyde reduced growth rate (*P* = 0.001) while the AA inclusion had no evidence of impact. Formaldehyde reduced feed bacterial content and altered fecal microbial communities (*P* < 0.05). Therefore, we conclude that the negative influence on growth was due to the impact on the fecal microbial community. Implications are that strategies for feed pathogen control need to take into account potential negative impacts on the gut microbial community.

## Introduction

In the swine industry, in-feed antibiotics have been used to control enteric infections and improve growth performance^1^. The use of growth promoting antibiotics has led to increased antibiotic resistance in gut bacteria^2^. In swine production systems there are considerable interest and effort in identifying feeding and management practices that maintain and improve production efficiency without the use of in-feed antibiotics. Formaldehyde-based feed additives can be used to reduce and or prevent bacterial contamination in animal feed^3^. According to the Food and Drug Administration’s federal register^4^ (#21 CFR 573.460), the food additive formaldehyde can be included in animal feed or ingredients to maintain complete feed and ingredients as *Salmonella* negative for up to 21 days. Formaldehyde-based products are widely used in the poultry industry for *Salmonella* control in feed^5^. Since the emergence of porcine epidemic diarrhea virus (PEDV) in the United States and demonstration that feed can be a vector for the transmission of the disease^6^ formaldehyde-based products have received attention as a potential method to reduce the risk of PEDV transmission in feed. Research demonstrating formaldehyde use in reducing PEDV infectivity in contaminated feed and ingredients has been successful^7,8^ and has led to an increased use of formaldehyde in pig feed. However, formaldehyde is known to produce reactions with numerous groups of amino acid residues of proteins that can lead to the formation of methylol groups, Schiff-bases, and methylene bridges amongst these residues^9^. Thus, inclusion in diets may reduce the small intestinal availability of dietary amino acids (AA) for pigs, which may negatively influence growth performance and nutrient utilization^10–12^. Likewise, information is lacking regarding formaldehyde inclusion in the diet of pigs and changes in gut microbiota which contributes to nutrient utilization. Therefore, a study was conducted in nursery pigs to determine if formaldehyde treatment would interact with dietary Lysine concentration and crystalline AA concentration and affect nursery pig growth performance, feed bacteria concentration, and fecal microbiota.

## Results

### Feed bacterial concentration

For diet bacterial concentrations, there was no evidence of difference (P > 0.10) for a crystalline AA × Formaldehyde interaction for any bacterial plate counts evaluated (Table 1). There was also no evidence of difference (*P* > 0.10) in bacterial plate counts evaluated between the low and high crystalline amino acid diets. Analysis of feed samples revealed that the control diet had greater (*P* = 0.027) aerobic plate counts compared to the other treatment diets and marginally significant greater (*P* = 0.062) *Enterobacteriaceae* counts. Formaldehyde inclusion in diets reduced (*P* < 0.05) *Enterobacteriaceae* and total coliform counts compared to diets that did not include formaldehyde.

**Table 1:** Effect of formaldehyde-treated diets and crystalline amino acid level on complete feed bacterial concentration and lysine content.

	Control	Low Crystalline AA		High Crystalline AA		Probability, *P* <			
		No formaldehyde	Formaldehyde	No formaldehyde	Formaldehyde	Control vs. Others	Crys AA × Formaldehyde	Low vs. High Crystalline	Formaldehyde
**Feed bacterial concentration** ^1^									
Aerobic plate count	5.5	5.0	4.9	5.0	4.5	0.027	0.422	0.330	0.242
SEM	0.23	0.23	0.36	0.23	0.36	—	—	—	—
Enterobacteriaceae count	4.1	3.7	1.9	4.2	1.2	0.062	0.455	0.879	0.014
SEM	0.29	0.29	1.02	0.29	1.02	—	—	—	—
Total coliform count	4.6	4.2	2.2	4.5	1.4	0.143	0.501	0.774	0.019
SEM	0.39	0.39	1.20	0.39	1.20	—	—	—	—
**Lysine content** ^2^									
Total lys	1.51	1.25	1.14	1.20	1.18	0.001	0.035	0.932	0.007
SEM	0.02	0.02	0.02	0.02	0.02	—	—	—	—
Total lys, % difference from calculated	−2.54	−2.00	−10.7	−3.32	−4.64	0.793	0.032	0.140	0.007
SEM	1.47	1.47	1.47	1.47	1.47	—	—	—	—

^a^Values represented in log_10_.

^b^Negative values indicate analyzed values were less than calculated values.

### Lysine analysis of the feed

As expected, the positive control diet contained a greater (1.51 ± 0.02; *P* = 0.001) amount of total Lys than the low or high crystalline amino acid diets with or without formaldehyde confirming that diets were formulated correctly (Table 1). Free lysine analysis confirmed that the crystalline AA were added correctly to the diets. A crystalline AA × Formaldehyde interaction was observed (*P* < 0.05) in analyzed diets for total Lys and total Lys percent difference from calculated values. This interaction occurred because formaldehyde inclusion in low crystalline AA diets reduced total Lys and had greater percent difference from calculated total Lys values than in the high crystalline AA diets. There was no evidence of difference between total Lys and percent difference from calculated total Lys values with or without the inclusion of formaldehyde in high crystalline AA diets.

### Growth performance of piglets

Overall (d 0 to 28), pigs fed the control diet had improved (*P* < 0.05) average daily gain (ADG), ending body weight (BW), and gain to feed ratio (G:F) compared to those fed other diets containing reduced Lys (Table 2). This confirmed that Lys was limiting in the diets for pigs fed below their Lys requirement. The application of formaldehyde to diets reduced (*P* < 0.05) ADG (513 ± 7.30 vs 519 ± 7.30 g/d) and ending BW (26.8 ± 0.280, 26.7 ± 0.280 kg) compared to not treating diets with formaldehyde. A Crystalline AA × formaldehyde interaction (*P* < 0.05) was observed for average daily feed intake (ADFI) and G:F. The interaction for ADFI occurred because treating diets with formaldehyde decreased ADFI for pigs fed diets with high crystalline AA inclusions, but did not influence ADFI for pigs fed low crystalline AA diets. The interaction for G:F was observed because treating diets with formaldehyde decreased G:F for pigs fed low crystalline AA diets but did not influence G:F for pigs fed high crystalline AA diets.

**Table 2:** Effect of formaldehyde-treated diets and crystalline amino acid level on nursery pig performance.

	Control^2^	Low crystalline AA^3^		High crystalline AA^3^		Probability, *P*<				
		No formaldehyde	Formaldehyde^4^	No formaldehyde	Formaldehyde	SEM	Control vs. Others	Crys AA × formaldehyde	Low vs. High crystalline	Formaldehyde
**d 0 to 28**										
ADG, g	601	542	513	543	519	7.30	0.001	0.757	0.637	0.001
ADFI, g	945^a,b^	927^b,c^	930^b,c^	960^a^	911^c^	12.41	0.921	0.020	0.526	0.036
G:F	0.636^a^	0.585^b^	0.553^d^	0.566^c^	0.567^c^	0.003	0.001	0.001	0.478	0.001
**BW, kg**										
d 0	12.2	12.2	12.3	12.2	12.2	0.120	0.530	0.278	0.142	0.139
d 28	29.1	27.4	26.8	27.5	26.7	0.280	0.001	0.713	0.931	0.001

^a,b,c,d^Means within same row with different superscripts differ (*P* < 0.05).

### Bacterial community structure

**Figure 1:**
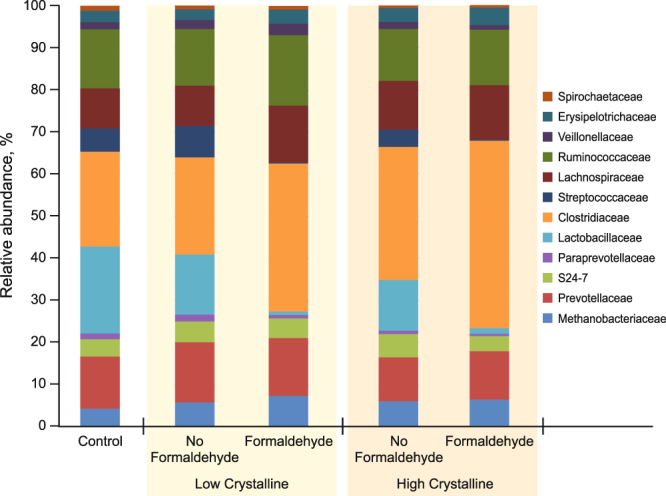
Bacterial abundance analysis among treatments groups evaluated at the family level. The graph exhibits the percent of each bacterial species that was detected.

At the phylum level, the most dominant phyla were *Firmicutes* (74.8%) and *Bacteroidetes* (15.6%); however, there were no effects of dietary treatment at the phylum level. At the family level, 49 families were identified, among which 12 families having greater than 1% of core sequences. There was no evidence of differences (*P* > 0.10) in bacterial abundances amongst the dietary treatments for *Methanobacteriaceae*, *Prevotellaceae*, *Lachnospiraceae*, or *Spirochaetaceae*. A crystalline AA × formaldehyde interaction (*P* = 0.003) was observed for Streptococcaceae abundances in the bacterial community of the gut, because pigs fed low crystalline AA diets had a more dramatic reduction in abundance when treated with formaldehyde compared to the high crystalline AA diets (Fig. 1). The treatment of diets with formaldehyde decreased (0.69 ± 0.17, 0.48 ± 0.17; *P* < 0.05) bacterial abundance for *Paraprevotellaceae* and *Lactobacillaceae* species, while formaldehyde treatment increased (27.5 ± 2.65, 35.5 ± 2.65; *P* < 0.05) *Clostridiaceae* and *Erysipelotrichaceae* species within the bacterial community of the gut. Pigs fed formaldehyde-treated diets had marginal significance (3.75 ± 0.53, 2.94 ± 0.53; *P* = 0.074) for lower percentages of S24-7 bacteria species than pigs fed non-formaldehyde treated diets. Pigs fed low crystalline AA diets had increased (*P* < 0.05) abundance of *Paraprevotellaceae*, *Lactobacilliaceae*, *Ruminococcaceae*, and *Veillonellaceae* bacterial species compared to high crystalline AA diets. Pigs fed high crystalline AA diets had increased (25.9 ± 2.65, 35.5 ± 2.65; *P* = 0.007) *Clostridiaceae* and tended (2.51 ± 0.33, 3.19 ± 0.33; *P* = 0.080) to have increased *Erysipelotrichaceae* bacterial species compared to pigs fed low crystalline AA diets. Treatment diets fed to lower lysine levels than the control had increased (*P* = 0.009) *Clostridiaceae* bacterial species, while *Paraprevotellaceae* species were marginally (*P* = 0.091) lower in these diets compared to the positive control.

## Discussion

Our results are in agreement with earlier research using formaldehyde to reduce bacterial load in complete feeds for poultry^13^ and swine^14,15^. The addition of formaldehyde to poultry diets reduces contamination from specific bacterial pathogens such as *Salmonella*^13^. This is consistent with our results that indicated formaldehyde reduced *Enterobacteriaceae* and total coliform count in feed.

The use of formaldehyde and the subsequent effect on animal performance has been more extensively investigated in poultry compared to swine. For example, one study evaluated the addition of a commercially available formaldehyde additive to broiler diets and it improved feed conversion ratio by 4.8%^16^. However in another study, chickens received 1 of 4 diets treated with either 0, 630, 1580 or 6,300 mg formaldehyde/kg feed^17^. A reduction in body weight was observed for chickens fed formaldehyde treated diets, regardless of level, compared to the untreated feed. In swine, more limited research has been conducted evaluating the effects of formaldehyde on single ingredients or the complete diet on bacterial concentrations and performance. One study evaluated the application of formaldehyde to spray-dried animal plasma prior to complete diet manufacturing, which reduced the bacterial concentration^14^. Pigs fed diets with 6% formaldehyde-treated animal plasma had improved ADG and ADFI compared to pigs fed control diets with untreated animal plasma. In a follow up study these researchers observed that formaldehyde application to the complete diet reduced bacterial concentration of the total diet compared to those not treated with formaldehyde, but growth performance was not improved^14^. This study is consistent with our study suggesting that treating the complete diet reduces growth performance. We believe that formaldehyde treatment of an ingredient that was a relatively low proportion of the diet (6%) did not affect the microbial diversity of the feed, however treating the complete diet did affect the microbial content of the feed and led to reduced growth performance.

Formaldehyde has the ability to produce reactions with numerous groups of AA, including Lys^9,18,19^. Thus, we hypothesized the form of AA supplementation (crystalline AA or intact AA in protein) maybe affected by formaldehyde may be affected differently as reported previously^20^. The reactions between formaldehyde and AA, especially Lys, could render these AA unavailable which could possibly alter growth performance. Our results suggest the effect was greater in the low crystalline AA diets which contained more intact protein as a source of amino acids. The reactions that occur between formaldehyde and Lys residues in proteins may explain why formaldehyde is reducing the amount of total and available Lys in the current studies and could alter protein utilization, thus explaining the reduction in performance. The growth performance data suggests that formaldehyde is affecting the growth performance of the pigs both through the nutritive content of the feed and by affecting the microbial community within the feed.

The gastrointestinal tract of pigs harbors beneficial commensal bacteria that play a role in regulating gene expression that can influence gut health and immune responses^21^. Advancements in sequencing techniques have allowed researchers to focus on sequencing specific regions within the 16S rRNA gene to reveal patterns in microbial composition of the pig gastrointestinal tract^22^. Holman *et al*.^23^ utilized a meta-analysis to determine the major types of commensal bacteria species that inhabit the gastrointestinal tract of pigs. The researchers observed that *Prevotella, Clostridium, Ruminococcus*, and *Lactobacillus* species were found in greater than 90% of fecal samples collected in those studies, which would be similar to the findings of the current study. However, little data has been generated in pigs documenting the effects dietary manipulations have on gut microbial diversity. One study had demonstrated dietary effects on weaned pig fecal microbial diversity^24^ and other researchers have evaluated macro minerals and in-feed antimicrobials and their effects on gut microbiota changes^25,26^. When looking at the relationship between gut microbiota and nutrient utilization, there is an established possible link in piglets where feed efficiency is directly related to gut microbial compositional differences with a higher relative abundance of beneficial bacteria such as *Clostridiales* and *Bacteroidetes*^10^. However, our study is the first to observe the effects of formaldehyde treatment of diets on nursery gut microbiota. More uniquely we have demonstrated a decrease in lactic acid bacteria species, specifically *Lactobacillaceae*, and the increase of *Clostridiaceae* species in fecal samples obtained from pigs fed formaldehyde-treated diets. This warrants further investigation to determine the short- and long-term effects this shift in commensal bacterial species has on gut health. It has been well documented that antimicrobial therapy plays a pivotal role in the pathogenesis of Clostridia infection in humans, particularly *Clostridium difficile* infection. Antimicrobials known to disrupt the indigenous microbiota of the hindgut (colon), allowing Clostridia to grow in high concentrations^27^. Thus, in our study we shifted the microbial population by reducing more desirable species and increasing species with potential negative side effects.

In summary, as expected the inclusion of formaldehyde reduced bacterial concentration in nursery pig feed. However, these studies have provided evidence that in nursery pigs the inclusion of formaldehyde in complete feeds has a negative impact on growth performance. In addition, the inclusion of formaldehyde altered fecal microbial populations with a reduction in *Lactobacillaceae* species but increase in *Clostridiaceae* species. Thus, implications of this research are that the effect of pathogen control needs to be balanced against potential negative influences on microbial populations.

## Materials and Methods

### Animals and study design

The Kansas State University Institutional Animal Care and Use Committee (IACUC # 3529) approved the protocol used in this experiment and all experiments were performed in accordance with the relevant guidelines and regulations. A total of 1,235 pigs (PIC 359× Gentiporc F25 initially 12.2 ± 0.12 kg) were used in a 28-d study. Pens of pigs were allotted to 1 of 5 dietary treatments based on average BW and location within barn with 19 to 22 pigs per pen (with a similar number of barrows and gilts in each pen) and 12 replications per treatment in a randomized complete block design. Dietary treatments were arranged in a (2 × 2) + 1 factorial. The diet formaldehyde (1000 ppm) treatment was done using a commercially available product (3.2 kg/tonne, Sal CURB, Kemin Industries, Inc., Des Moines, IA) and crystalline AA inclusion were low and high. A positive control was used in the experiment to represent diets that met the assumed standardized ileal digestible (SID) Lys requirement estimate for pigs used in this study. Other treatment diets were formulated to be 80% of the SID Lys concentration in the positive control diet. Pigs and feeders were weighed to determine average daily gain (ADG), average daily feed intake (ADFI), and gain to feed ratio (G:F). The study was conducted at typical standard pig production facility in Iowa. Each pen (2.44 × 5.64 m) was equipped with a 4-hole, dry-self feeder and a pan waterer to provide ad libitum access to feed and water.

### Feed sample collection

Feed samples were collected directly from each individual batch of feed in 5 spaced sub-samples by passing sterile Whirl-Pak (Nasco, Ft. Atkinson, WI) through the stream of feed as it was emptied from load-out bin into the feed delivery truck. Feed samples were also collected directly from 6 different feeders for each dietary treatment and placed in sterile Whirl-Pak bags to represent farm samples. Both mill and farm samples were pooled within collection location and transported to the Department of Diagnostic Medicine/Pathobiology, College of Veterinary Medicine, Kansas State University for feed bacterial enumeration analysis.

### Feed bacteria enumeration

Feed samples were evaluated for bacterial counts using specialized plates (3 M Petri film 3 M Microbiology, St. Paul, MN) with each plate selecting for certain organisms. The specific organisms being detected for this experiment were: total coliforms (TC), aerobic plate counts (APC), and *Enterobacteriaceae* (EB). One gram of feed sample was diluted in 10 mL of phosphate buffered saline (PBS) tube and vortexed to make a uniform suspension before serially diluting the feed suspension to achieve 10^0^, 10^−1^, 10^−2^, and 10^−3^ concentrations. With a sterile pipette, 1 mL of feed sample at each dilution was placed in the center of the Petrifilm in triplicates for each plate type. Dilutions were vortexed before plating to ensure equal distribution of feed inoculum. The sample inoculum was uniformly distributed over a 20 cm^2^ circular plate area. Total coliform, *Enterobacteriaceae*, and Aerobic plates were incubated for 48, 24, and 48 hrs, respectively. After incubation, colony number, colony morphology, gas production, and acidification for each plate was performed using a commercial plate reader (3 M Petrifilm, St. Paul, MN). Counting ranges were 50 to 150 colonies for coliform plates, 15 to 100 colonies for *Enterobacteriaceae* plates, and 25 to 250 colonies aerobic count plates. Colony counts were expressed as colony forming units per g of feed sample (cfu/g) and bacterial counts were expressed as an average of 2 separate runs ran in duplicate with a different feed sample.

### Fecal sample collection

Fecal samples were collected via rectal massage into individual sterile bags from 6 randomly selected pigs on d 0 and from 3 randomly selected pigs per pen on d 28. Samples were stored at 4 °C and transported to Kansas State University where d 28 samples were pooled within pen into individual samples to represent 6 baseline samples and 12 samples per treatment. Samples were stored at −80 °C until transportation to the University of Nebraska-Lincoln for bacterial community analysis.

### DNA Extraction and PCR Amplification

Fecal DNA (6 from baseline, 12 per treatment) were isolated from approximately 100 mg samples using a commercially available kit (Mag-Bind Soil DNA Kit; Omega Bio-tek, Inc., Norcross, GA) according to the manufacturer’s protocol with the following modifications: the raw sample was transferred to a 2 mL sterile Safe-Lock tube (Eppendorf, Hauppauge, NY) with 0.3 g of acid washed beads (Scientific Asset Management, Basking Ridge, NJ) and 300 µL of SLX-Mlus Buffer, followed by bead-beating at frequency of 20 for 10 min (QIAGEN Inc., Valencia, CA); after samples were mixed with RNase A and DS Buffer, samples were incubated in a 90 °C water bath for 8 min and occasionally vortexed; the samples were centrifuged at a speed of 5,000 $\times$ *g* for 10 min and the supernatant was transferred; finally, DNA was eluted with 130 µL of elution buffer at the last step.

The V4 region of the 16S rRNA gene specific to the eubacterial communities was amplified from the extracted DNA samples^28^. A PCR reaction (20 µL) consisted of 2 µL of template DNA, 0.5 µL each of both forward and reverse 16S rRNA V4 primers (final concentration 10.0 µM; Integrated DNA Technologies, Coralville, IA), 0.5 µL of Terra PCR Direct Polymerase Mix (Clontech Laboratories Inc., Mountain View, CA), 10 µL of 2× Terra PCR Direct Buffer (Clontech Laboratories Inc., Mountain View, CA) and 6.5 µL of nuclease-free water (Hoefer Inc., Holliston, MA). Using a Veriti 96-well thermocycler (Life Technologies, Carlsbad, CA), the amplification was performed at 98 °C for 3 min, followed by 25 cycles of 30 s at 98 °C, 30 s at 55 °C, and 45 s at 68 °C, with a final elongation stage of 4 min at 68 °C. The amplified PCR products were tested by agarose gel electrophoresis (5 µL PCR product, 1.5% agarose gel) at 100 V for 60 min for size verification and to confirm amplification.

### Preparation of 16S rRNA Library and sequencing

From each sample, 10 μL of the PCR product was pooled together and mixed. The pooled 16S rRNA gene library was column purified using PCR cleanup procedure (DNA, RNA, and protein purification; Clontech Laboratories, Mountain View, CA) and eluted into 40 µL. Subsequently, the Pippin Prep (Sage Science, Beverly, MA) was used to remove any spurious PCR fragments from the purified concentrated library. Finally, sequencing was performed using the Illumina MiSeq platform (Illumina, Santa Clara, CA) according to the manufacturer’s protocol.

### Bacterial Community Analysis

The raw reads were demultiplexed using the Quantitative Insights Into Microbial Ecology program (QIIME^29^) and run through a quality control described by Anderson *et al*.^30^. Reads were trimmed to a fixed length of 251 bp using Mothur^31^ and the FASTX-TOOLKIT^32^, followed by identification of operational taxonomic unit (OTU) based on 97% similarity using UPARSE pipeline^33^. The generated OTU sequences were aligned using Ribosomal Database Project (http://pyro.cme.msu.edu). Taxonomic classification was performed using QIIME and the Green Genes database^32^ (version 13_8). The OTUs belonging to the phylum *Cyanobacteria* were removed as these are from dietary source. Single sequences that may be generated from sequencing error, were removed from the data. A core set of 854 OTUs (42.3% of the original OTU table), presenting in at least 75% of the samples, was identified for further analysis.

### Fecal Microbial Diversity

Abundance in different taxonomy levels in bacterial community structure (β-diversity) were evaluated using normalized unweighted UniFrac distance matrices, with an OUT defined at an identity cut-off at 97% (14,163). The unweighted UniFrac distance matrices were used as input for a multivariate analysis using the Fathom Toolbox for MATLAB^34^.

### Statistical Analyses

The AA content as represented as the Lysine concentration of the feed was evaluated using the analyzed value and the percent difference of analyzed values from expected formulated values. Feed bacterial concentration was analyzed by converting these values to log_10_. Pig growth performance as represented by ADG, ADFI and G:F were analyzed as a randomized complete block design using analysis of variance based on the assumption of normal distribution and heterogeneous variance. Pen was considered the experimental unit for growth performance and bacterial community analysis. Bacterial community analysis data were analyzed as OTU abundances at the family level. Fecal microbial diversity and the observed core OTU, Chao 1 and Shannon index from 10 subsampling events per fecal sample. Treatment was considered the fixed effect and a random effect of block. Residuals for each response criteria were evaluated for evidence of lack of normality and heterogeneous variance. Feed bacterial concentration had evidence of lack of normality and thus was log transformed and heterogeneous variance accounted for in the statistical model. No evidence for heterogeneous variance was noted for other criteria and thus variance was reported as pooled standard error of the mean. Pre-planned contrasts were utilized to compare the interaction between crystalline AA level and formaldehyde inclusion, the main effects of formaldehyde inclusion or crystalline AA level, and crystalline AA inclusion compared to the positive control. All data were analyzed using the GLIMMIX procedure of SAS 9.4^35^. Results are reported as lsmeans +/− the standard error of the mean and were considered significant at *P* ≤ 0.05 and marginally significant at *P* > 0.05 and *P* ≤ 0.10.
